# Monitoring Bioindication of Plankton through the Analysis of the Fourier Spectra of the Underwater Digital Holographic Sensor Data

**DOI:** 10.3390/s24072370

**Published:** 2024-04-08

**Authors:** Victor Dyomin, Alexandra Davydova, Nikolay Kirillov, Oksana Kondratova, Yuri Morgalev, Sergey Morgalev, Tamara Morgaleva, Igor Polovtsev

**Affiliations:** 1 Laboratory for Radiophysical and Optical Methods of Environmental Research, National Research Tomsk State University, 36 Lenin Avenue, 634050 Tomsk, Russia; dyomin@mail.tsu.ru (V.D.); knsnik@gmail.com (N.K.); polovcev_i@mail.ru (I.P.); 2 Center for Biotesting of Nanotechnologies and Nanomaterials Safety, National Research Tomsk State University, 36 Lenin Avenue, 634050 Tomsk, Russia; kov-2710@yandex.ru (O.K.); yu.morgalev@gmail.com (Y.M.); s.morgalev@gmail.com (S.M.); tg.morgaleva@gmail.com (T.M.)

**Keywords:** bioindication, monitoring, plankton, underwater digital holographic camera, behavioral characteristics

## Abstract

The study presents a bioindication complex and a technology of the experiment based on a submersible digital holographic camera with advanced monitoring capabilities for the study of plankton and its behavioral characteristics in situ. Additional mechanical and software options expand the capabilities of the digital holographic camera, thus making it possible to adapt the depth of the holographing scene to the parameters of the plankton habitat, perform automatic registration of the “zero” frame and automatic calibration, and carry out natural experiments with plankton photostimulation. The paper considers the results of a long-term digital holographic experiment on the biotesting of the water area in Arctic latitudes. It shows additional possibilities arising during the spectral processing of long time series of plankton parameters obtained during monitoring measurements by a submersible digital holographic camera. In particular, information on the rhythmic components of the ecosystem and behavioral characteristics of plankton, which can be used as a marker of the ecosystem well-being disturbance, is thus obtained.

## 1. Introduction

The ubiquity of plankton, its ecosystem significance, close relationship with the environment and its quick response to pollution indicate the prospects for its use as a biosensing organism for the further bioindication of pollution.

All members of the biological community are connected to each other and to the surrounding abiotic environment by various types of bonds. The ecosystem is therefore a functional system that includes a community of living organisms (biocenosis) and their habitat with a set structure of bonds. Accordingly, the ecosystem function (or ecosystem characteristic) describes the dynamics of the ecosystem as well as its development over time. The ecosystem function can be presented, for example, as the time sequence of a concentration of individuals of a certain taxon in a certain volume, the rate of response to an irritant, the species ratio of individuals in the system, etc. Disaster in this case is a mathematical feature in the behavior of the ecosystem function, which the system crosses before shifting into a considerably different state. Note that due to the high connectivity of biocenosis organisms, the ecosystem function can be built by studying the dynamics of one, but the most important biological link—dominant species.

Environmental disaster is not something that happens instantaneously. It is always developed with some hidden or obvious manifestations—precursors. In fact, unusual behavior, abnormal behavioral responses or the violation of the equilibrium “predator-prey” ratio [1,2] in the biodiversity are the bioindicators of violation of the stable structure of links characteristic of the ecological well-being, which serve the precursors that increase the possibility of a disaster.

The in situ monitoring of precursors allows finding the peculiarities of the system behavior to detect the pre-disaster states. The use of the behavior of higher animals as the precursors of disasters is well known. In this respect, plankton is an ideal object because of its high dependence on the medium and low response threshold. This special role of the free-living plankton is determined by its place at the very beginning of the food chain as well as by the filtering mechanism of its nutrition [3,4]. This feature is used in the method of biotesting the water quality, known as the daphnia test, which is based on the analysis of the behavior of specially cultivated plankters [5,6,7].

Moreover, it is necessary that the ecosystem function is deterministic, and then the monitoring starting moment is not critical here. It is also important that at the time of the beginning of a number of observations, the ecosystem is stable with respect to at least deterministic species. 

This paper presents the results of long-term monitoring measurements using a hydrobiological complex based on a submersible digital holographic camera (DHC) performed from 23 July to 24 September 2022. It also suggests a new method of processing data obtained by an underwater holographic sensor using the Fourier spectrum of the time series of measured data. This method makes it possible to identify regular information against the background of random factors that do not affect the quality of life of biocenosis but do affect the number of inhabitants. The DHC was used to study the collective phenomena in the biotic community, including plankton migrations that form the rhythm of the ecosystem characteristics. The possibility of building a bioindication system to assess the risks [8,9] of undesirable development of the situation in the marine environment was studied using the example of a bioindication station that monitors the free-living plankton. At the same time, in accordance with the principle of bioindication, we replace the totality of all environmental parameters affecting the ecosystem function with the concept of environmental comfort that ensures all behavioral responses of plankton. 

Digital underwater holography [10,11,12,13,14] is the most effective and informative method for plankton study in the habitat, since it has the necessary set of functions and parameters that are critical for in situ studies. It ensures the automatic digital focusing, makes it possible to control the size of the data sample, and transmits digital holograms over communication lines.

In our works [10,11,15,16], we presented several versions of submersible digital holographic cameras (DHCs), as well as the DHC technology, which includes an automated recording of a medium hologram, the reconstruction of images of each plankton individual, and the determination of plankton size, shape, and concentration, as well as its classification. The object of the study using the DHC technology is zooplankton, while the subject of the study is its geometric, ecosystem and behavioral characteristics. The possibility of recording all suspended particles of the measuring volume on one hologram and their subsequent study based on reconstructed holographic images, as well as the possibility of monitoring measurements, i.e., the registration of time sequences of holograms, allow the DHC technology to study the ecosystem and behavioral characteristics regarding their differences in response efficiency, depending on the task personified in relation to particular groups and even individuals.

At the same time, it is important to be able to adapt the functions and parameters of the DHC to the features of the habitat and the addressed task. To interpret the time series of plankton indicators, the present work uses the spectral analysis, which makes it possible to obtain information on the rhythm of plankton processes (periodicity of the ecosystem function) and, in case of its disturbance, to make conclusions on the disturbance of the ecological state of the ecosystem at early stages.

## 2. Equipment and DHC Technology for Bioindication

### 2.1. Equipment and DHC Technology

The use of submersible holographic cameras makes it possible to record information on all suspended particles of the measuring volume in one hologram frame, which allows solving a wide variety of problems for the registration, counting and classification of plankton organisms in situ. 

**Figure 1:**
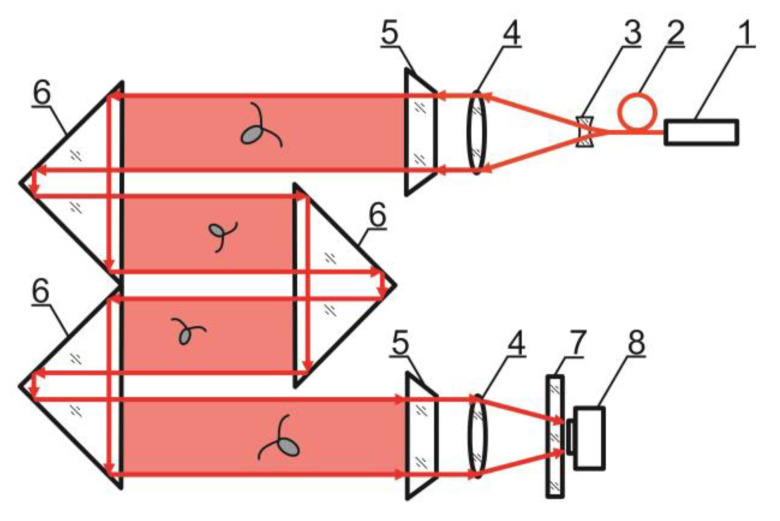
DHC scheme: 1—laser diodes with fiber output, 2—optical multiplexer, 3—beam expander, 4—lenses, 5—portholes, 6—prisms forming the working volume, marked red, 7—selective filter, 8—CCD/CMOS camera.

The in-line scheme of digital hologram recording implemented in the DHC (TSU, Tomsk, Russia) is shown in Figure 1. 

The DHC has several coherent light sources representing laser diodes of different wavelengths with fiber outputs combined into a multiplexer. A single-mode fiber with radiation, which plankton is indifferent to, is always used for hologram recording. In other words, the behavior of individuals under this illumination is similar to their natural behavior. 

An interference pattern of the reference wave (radiation that passed by the particles) and the object wave (radiation scattered on the particles) is formed as a result of laser radiation illumination of the studied volume of the medium containing particles. 

The camera registers this interference pattern as the two-dimensional array of discrete quantified intensity values, which is a digital hologram of the volume. The hologram is transmitted to the computer memory and then used as the initial field distribution for numerical layer-by-layer reconstruction of the holographic image of the medium volume with particles. In this case, with the help of the developed software, information on each particle (plankton individual, settling non-living particle, oil drop, gas bubble, etc.) contained in the recorded volume of the water medium is reproduced—size, shape, location in space, taxon, and when registering the time sequence of holograms—motion parameters. To obtain information on the studied particles from a digital hologram, the following steps should be taken:

1. Pre-processing of the digital hologram to remove background noise by subtracting the “zero” hologram;
2. Reconstruction of the specified number of images of cross-sections of the studied volume with particles from a digital hologram by convolution method [17,18,19];
3. Constructing a 2D volume display by compiling the sharpest images of particles into one image (a boundary intensity jump serves the criterion for choosing a sharp image of a particle [20]);
4. Automatic recognition and classification of particles according to their morphological characteristics [11].

Almost all processes—from controlling the hologram recording modes to reconstructing the holographic images and obtaining information on particles—are automated and represent the so-called DHC technology [10]. 

**Figure 2:**
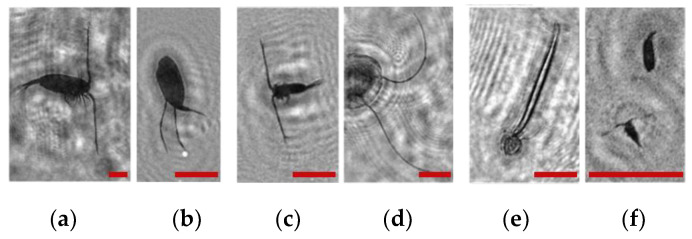
Holographic images of plankton particles located in different parts of the DHC working volume ((**a**,**b**)—particles in part I (Figure 3b), (**c**,**d**)—in part II (Figure 3b), (**e**,**f**)—in part IV (Figure 3b)), obtained using the DHC in the natural conditions of the Arctic expedition. Size of the scale ruler in all images—500 µm.

**Figure 3:**
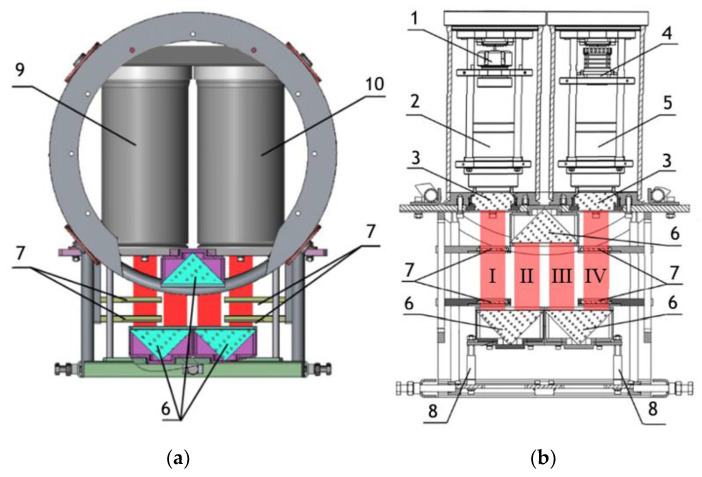
(**a**) General layout of the DHC for plankton monitoring, (**b**) layout of nodes. 1—laser diode, 2—beam expander, 3—portholes, 4—CMOS camera, 5—optical system for optical radiation receiving, 6—mirror-prism system to form a measuring channel in the medium (working volume), 7—calibers, 8—replaceable rods, 9—lighting module, 10—recording module. The red color marks the working volume (studied medium volume). I, II, III, IV—parts of the working volume separated by prisms.

Note that this DHC (Figure 1) includes a lens to increase the field of view and a mirror prism system to reduce (fold) the dimensions of the device as opposed to the traditional in-line scheme (Gabor scheme). This “folded” scheme is used in all modern DHC modifications. At the same time, in some tasks, we remove the receiving lens from the scheme in order to obtain non-scaled holographic images of particles (Figure 2) [11]. The appearance and internal layout of the DHC are shown in Figure 3.

The maximum DHC working volume shown in red in Figure 3b is 0.48 dm^3^ and has a length of 694 mm. Under the conditions of turbidity or turbulence of the medium, the length of the working volume for this modification can be set equal to 294, 394, 494, 594, or 694 mm with the use of replaceable rods (8). It is possible to identify plankton particles with a size varying from 100 to 10,000 μm located in pure water in the entire range of the specified distances (working volume lengths) (Figure 2). The medium may negatively affect this indicator and then will cause the need to reduce the working volume [15].

The DHC design implies a parallel beam illuminating the working volume, as shown in Figure 3, so there is almost no loss of light energy at a distance due to divergence. Much more important is the loss of information due to the interaction of the light field with medium inhomogeneities. This causes some restrictions to be imposed on the working volume length to ensure the image quality within the entire working volume. Thus, for clean water without turbulence, the length of the working volume is 500 mm; for turbid water, it is 50 mm. In this case, the size of the individual with normal details of the structure is ~100 μm along the entire length of the working volume, as shown in Figure 2.

The device has four calibers (7) for calibration by magnification. The caliber is an opaque square particle with a size of 500 × 500 µm, which is placed on a glass plate by a photolithography method. The glass plate is placed into the metal frame to be safely fixed in the working volume. Such a design ensures the autocalibration by magnification in the same medium as the studied particles and eliminates the need to take into account the optical properties of the medium. 

The overall dimensions of the DHC are 581 × 290.5 × 450 mm, and the weight is 23 kg, which makes it easy to use and transport. The power supply of the device operates at 12 V and consumes 20 W of power. The device is equipped with an optical fiber system of laser diodes with different wavelengths of radiation, which makes it possible to conduct various studies of the water medium, including the fluorescence excitation of phytoplankton chlorophyll (wavelength—0.45 μm) and attracting light to excite the phototropic response of zooplankton (wavelength—0.52 μm). A single-mode laser diode is used to register digital holograms at a wavelength of 0.66 ± 0.05 μm. 

The Mako G-507 CMOS camera (Allied Vision, Stadtroda, Germany [21]) equipped with a Sony DMX264 (Sony Group Corporation, Tokyo, Japan) matrix (2464 × 2056 pixels with a pixel size of 3.45 × 3.45 μm) is used for digital hologram recording. The system has a high data transfer rate over the Ethernet channel—up to 1 Gbps, which ensures continuous frame recording at a frequency of up to 25 fps. 

During field studies, the DHC and all the necessary additional medium sensors serve the components of a probe and are connected to an information unit forming a single hydrobiological probe—the DHC probe. The operation modes of the probe depend on the addressed research task in natural conditions. The most common mode is when every hour, the hydrobiological probe records the data on plankton concentration in the DHC working volume and the factors (characteristics) of the medium: pressure, temperature, conductivity, oxygen content, upper illumination, etc. The DHC probe can also operate in a vertical sensing mode with a fairly diverse sinking velocity ranging from 0.1 to 1.0 m/s. When a depth profile is formed, the discreteness of readings provided by the device is ~1 m, which makes it possible to obtain up to 20 depth profiles per day in a photic layer. 

The above DHC with additional mechanical options and the DHC technology modified with additional software provide the following advanced monitoring measurement capabilities for bioindication, thus making it different from other similar developments [14,22,23] in this field:

- Adjustment of the registered space depth for further adaptation to the medium conditions;
- Recording of a “zero” hologram for the correction of photometric noise;
- Autocalibration by magnification for the correction of large-scale distortions and reliable determination of dimensions and coordinates in reconstructed holographic images of particles without the need to determine the optical parameters of the medium;
- Additional light sources for plankton photostimulation, whose operation is synchronized with the CMOS camera and the laser diode recording a hologram;
- Possibility of controlling the hologram samples presented for analysis.

### 2.2. The Bioindication Informativity

The above features make it possible to obtain a representative sample of data on plankton concentration in the studied DHC volume as well as in real time due to long-term monitoring measurements in situ, and at a real station, despite the limited scale of the measuring space, including with the use of various impacts to stimulate plankton manifestations. Once the images of plankton particles are reconstructed from holograms and their concentration is estimated, the results presented as the time series of concentration form a monitoring series of the ecosystem function values both according to natural and, if necessary, according to photostimulated states of plankton. In this context, the device that registers plankton and measures its characteristics is called a digital holographic sensor. 

The object of the DHC study is free-living (autochthonous) zooplankton and ecosystem functions based on plankton concentration on a certain spatial scale (DHC working volume) but representing the long monitoring series of measurements. Due to the different rate of plankton community interaction with environmental factors [5,6,13,14,24,25,26,27,28,29,30,31,32], including the appearance of pollutants, it is possible to use the ecosystem functions that are different in terms of responsiveness. 

As mentioned above, this paper presents the results of natural measurements of plankton variability during the long-term moorage of the DHC-based hydrobiological complex with remote data transmission. Note that the DHC control signals and the measurement results were generated, received and processed via mobile communication with an operator being at a distance of more than 3000 km.

During the experiment, the concentration of individuals was measured in the working volume of the DHC fixed in space, which in general is not a constant but rather depends on the time of day, season and various environmental factors. Therefore, the measurements of plankton concentration reveal its spatiotemporal inhomogeneity. For various reasons, this inhomogeneity has rhythmic manifestations synchronized by the external influence. 

The most popular of the rhythmic manifestations is the circadian rhythm synchronized by sunlight and characteristic for all living beings [33]. This rhythm lasts ~24 h. 

The stability of the circadian rhythm in the living system indicates its well-being. Conversely, any instability sooner or later leads to an environmental disaster [34,35,36,37,38].

This study demonstrates the use of the underwater digital holography to build an ecosystem function in the form of a time series of measurements of plankton concentration in the DHC working volume, which can be used for the bioindication of the water area. The bioindication informativity of such an ecosystem function is related to the circadian rhythm of plankton. In this work, we use the Fourier analysis of time series of data obtained by an underwater holographic sensor, which allows establishing this rhythm and detecting the response of biosensoric organisms to pollution. Thus, the paper proposes a new method for processing data obtained using the underwater holographic sensor. 

## 3. Natural Marine Bioindication Experiment and Processing of Results

### 3.1. Monitoring of Plankton Concentration

The experimental material used in this work was obtained from the in situ monitoring of plankton concentration in water areas, with the parallel formation of its time series, as well as the calculation of other ecosystem functions in field conditions. This study considers only the issues related to the analysis of the time series of plankton concentration in a fixed working volume of the DHC.

**Figure 4:**
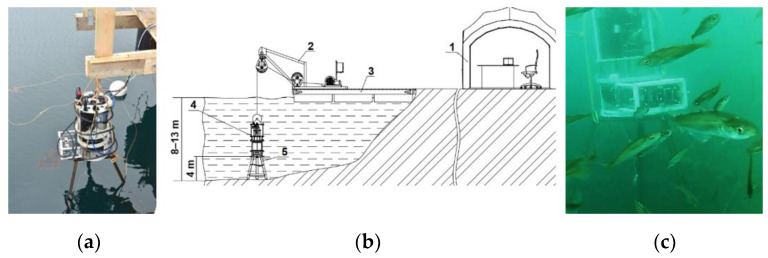
Moorage place of the DHC FOCL probe at Dalnye Zelentsy, geographical coordinates N 69°7′7.9″ E 36°4′10.6″: DHC FOCL probe before submergence (**a**), site layout plan and installation diagram (**b**), DHC FOCL probe fixed at the moorage place (**c**). 1—operator’s post at the onshore research station, 2—FOCL winch, 3—pontoon platform, 4—DHC FOCL probe, 5—bottom station.

A long-term monitoring experiment was performed in the Barents Sea, in the water area of the Zelenetskaya Bay, Russia, at the experimental training site of the Murmansk Marine Biological Institute of the Russian Academy of Sciences (MMBI RAS). The expedition lasted from 23 July to 24 September 2022. During this time, we managed to register a time series of measurements of plankton concentration in the DHC working volume during 32 days, starting from August 6. The station was located at a point with the following geographical coordinates: N 69°7′7.9″ E 36°4′10.6″. The depth of the water area at the moorage place at the middle point of tide was 9 m, and the fixed position of the station relative to the bottom was 4 m (Figure 4).

The point with the best ecosystem functions was chosen for the location of the probe based on preliminary reconnaissance works. The in situ data (digital holograms and measurements from other sensors) were transmitted to the central processor via a fiber-optic communication line for further computing. Therefore, the hydrobiological probe in this modification is called the DHC FOCL probe. 

**Figure 5:**
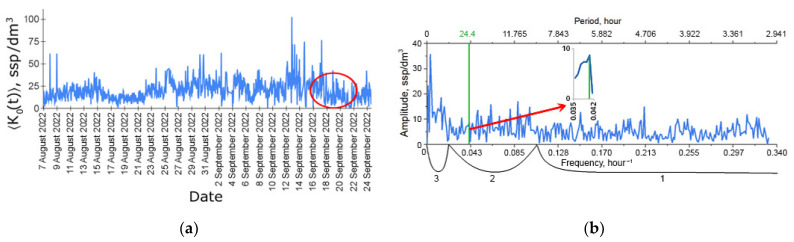
Time series of zooplankton concentration in the DHC working volume (**a**) and the Fourier amplitude spectrum (**b**) of this series during the moorage period used for analysis in this work. The green line corresponds to the frequency of the circadian rhythm (~1/24 h^−1^). The red circle in (**a**) shows the measurements obtained during the introduction of the indicative pollutant. (**b**) shows the ultradian rhythm range—1, diurnal rhythm range—2, seasonal rhythm range—3.

The simplest ecosystem function—the time series $\langle {К}_{0}(t)\rangle$ of plankton concentration in the DHC working volume—is presented in Figure 5a. Note that hereinafter, the index 0 means that the time series is considered with respect to all plankton species registered by the DHC without their specification. The averaging sign shows that several holograms, in this case *N* = 25, with an interval of 1 s between holograms, were used to form a single concentration reading. The concentration readings were made each hour for 32 days. 

Experimental data of the DHC working volume concentration measurements are presented as the mean ± $t_{0.05,\Lambda \;}\frac{SD}{\sqrt{\Lambda }}$. Here, $t_{0.05,\Lambda }$—Student’s coefficients for the significance level *p* ≤ 0.05 and the number of samples Λ, and SD—normal deviation. When forming a sample for averaging $\langle {К}_{0}(t)\rangle$, Λ = *N* = 25 holograms of the working volume $V_{W}$ were used to form an average reading over 1 h. On the other hand, Λ *= M*—number of hourly readings used to form one rhythm amplitude sample per day. *N* is determined at the stage of the natural experiment planning, thus linking the plankton concentration indicators in the water area, the size of the measuring volume and the volume of water having a representative value. The term “measuring volume” is applied to the number $V_{M}\left(1\right)=V_{W}\times N$ for hourly readings, and $V_{M}\left(48\right)=V_{M}\left(1\right)\times 48$—for daily readings, if a time series sample lasting M = 48 h is used for one reading of the ecosystem function per day. The concentration data are calculated to a volume of 1 dm^3^, since they are conveniently converted to 1 m^3^ (adopted in limnology and oceanology to characterize plankton concentration) by multiplying by 1000. 

### 3.2. Fourier Amplitude—Spectrum and Rhythms

The amplitude–frequency spectrum obtained by the discrete Fourier transform of the entire time series in Figure 5a is shown in Figure 5b. This information is required for qualitative spectral analysis, which makes it possible to establish the presence of characteristic frequencies—rhythms (explicit lines) in $\langle {К}_{0}(t)\rangle$ observed during the entire moorage period. This means that the number of data (registered holograms) used to detect a particular rhythm throughout the series is *N* × 24 × 32 = 19,200 holograms. 

For convenience of interpretation, the spectrum of zooplankton rhythms can be divided into the following ranges:

- Intra-day (ultradian) spectra, whose scale of changes is measured in hours, length—1/10 h^−1^–1/2 h^−1^;
- Diurnal spectra, whose scale of changes is measured in tens of hours, length—1/50 ^−1^–1/10 h^−1^;
- Seasonal and synoptic (infradian) spectra, whose scale of changes is measured in months, seasons, years, length—from 1/50 h^−1^.

**Figure 6:**
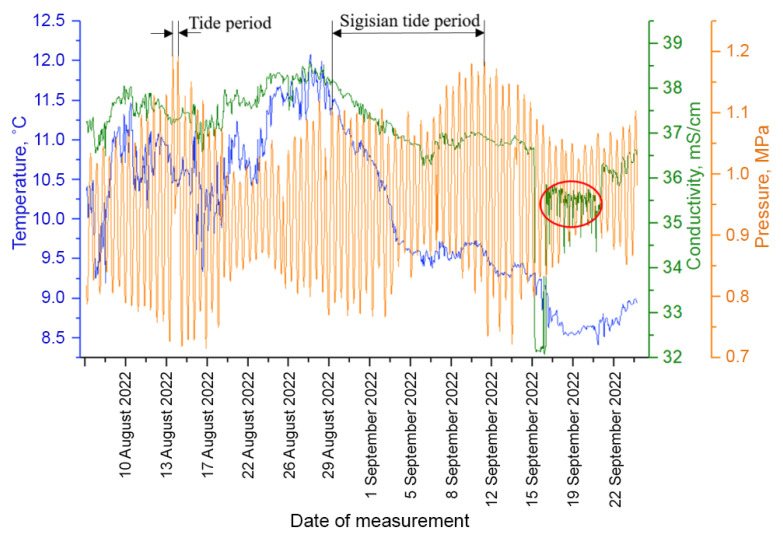
Time series of measurements of environmental factors at the moorage place, accompanying the measurements of plankton. The red circle indicates the contamination period.

Plankton measurements were followed by the measurements of media characteristics. The flow of this data is formed into the time series of hydrophysical parameters. Figure 6 shows an example of the time series of environmental factors registered and used for analysis in the present work.

A salt solution was dripped into the measuring zone to simulate a short-term impact on the medium parameters at the station. A saturated salt solution with a salt concentration of 300 g/L was used for supply with the rate of 20 L per day. NaCl is a harmless pollutant (indicator impurity) if its impact is not very long, for example, 5–10 days. A tank with table salt in the form of a perforated tube surrounding the probe was placed above the working volume for “contamination”. This allowed a “pollutant” to be injected locally into the DHC working volume and the adjacent zone without affecting the extensive biocenosis. At the same time, it was possible to control the parameters of the medium using an electrical conductivity sensor. The part of the data coinciding in time with the solution supply is marked with the red circle on the monitoring charts. 

One of the possible ecosystem functions—the series of amplitudes of the circadian rhythm recorded on the *j* day is quite interesting for this study. This function is defined as the maximum oscillation amplitude of plankton concentration in the DHC working volume close to the frequency $\sim \frac{1}{24\;}h^{-1}$:

(1) $${I_{c}}^{0}\left(j\right)=max\sqrt{{a_{c}\left(j\right)}^{2}+{b_{c}\left(j\right)}^{2}}$$

where $a_{c}\left(j\right)\;$ and $b_{c}\left(j\right)$—Fourier sine and cosine components of the circadian rhythm (*c*—circadian) obtained during the Fourier transform of the monitoring sequence of temporal plankton concentration measurements corresponding to day number *j*. 

At the same time, to calculate ${I_{c}}^{0}\left(j\right)$, we accept *M* = 48 that satisfies the Nyquist theorem, and to calculate ${I_{c}}^{0}\left(j\right)$, we “cut” a number of values $\langle {К}_{0}(t)\rangle$ for times

$\left(j-1\right)\cdot 24\;\left(h\right)+12\;(h)<t<\left(j+1\right)\cdot 24\;(h)+12\;(h)$. Superscript 0 means that all plankton taxa are taken into account.

The function $\;{I_{c}}^{0}\left(j\right)$ is the ecosystem function derived from processing the time series of experimental data on plankton concentration in the DHC working volume, which is used in the present work for bioindication purposes. The confidence interval of the hourly readings can be considered approximately constant and equal to

(2) $$∆{К}_{0}\approx t_{0.05,N}\frac{SD}{\sqrt{N}},$$

where SD—normal deviation of the concentration $\langle {К}_{0}(t)\rangle$ registered during the formation of the hourly readings. 

Considering that $t_{0,05,25}\approx \;t_{0,05,48}\approx 2$ and at Λ=*М* = 48, $\sqrt{\Lambda }\approx 7$ can be written to estimate the confidence interval of the circadian rhythm amplitude at *M* = 48:

(3) $$∆\;{I_{c}}^{0}\approx \frac{∆{К}_{0}}{7},$$

This ratio shows that if the circadian rhythm is determined with the duration of 48 h, then the Fourier transform averaging allows reducing the measurement error of its parameters by seven times. 

## 4. Results of Bioindication Experiment and Their Discussion

The above fixed moorage place of the hardware and software system—the DHC FOCL probe—ensures real-time observations of plankton and the environment. In fact, this refers to a stationary bioindication station that performs continuous monitoring at one point in the water area. Here, in this section, the time series data shown in Figure 5 are interpreted.

### 4.1. Biodiversity of Zooplankton at the Moorage Place

The samples taken by the net method with further processing of results in the laboratory were taken as the background data to characterize biodiversity. In accordance with the conclusion of the Murmansk Marine Biological Institute RAS, 34 taxonomic units of zooplankton were found in the fauna of zooplankton in the water area. The zooplankton concentration ranged from 5270 to 21,277 ssp/m^3^ depending on the place and time of sampling, making on average 11,060 ± 1811 ssp/m^3^. Copepoda dominated in terms of abundance and biomass, among which *Oithona similis* and nauplii Copepoda prevailed. Table 1 shows data on catching and holographic survey. 

In this work, we do not compare these data with data obtained using the DHC. We performed similar studies during the Arctic Expedition in 2020, and the error of the data obtained using the DHC was about 30% [11]. Table 1 shows that Copepoda organisms prevail in the medium, and their number is about 66.3–90.6%; therefore, the total number of plankton can be used to assess the rhythm of zooplankton (Figure 5), and this rhythm will be determined by the representatives of Copepoda. 

The results of the biodiversity analysis shown in Table 1 demonstrate that the organisms belonging to the Copepoda taxon determine the rhythm of zooplankton of the water area in Figure 5.

The reasons for the uneven background in the images in Table 1 include a small-scale turbulence of the medium and the twin-image effect typical for the used in-line holography scheme [15,39,40,41,42]. The first reason causes the need to select a moorage place and adjust the length of the recorded volume. The twin-image effect is reduced using additional numerical processing [40,42]. 

### 4.2. The Water Area Rhythm

Table 2 shows a list of rhythms from the spectrum in Figure 5b, which were determined as statistically significant based on the results of processing the time series in the Statistica 10 standard software (StatSoft Ink., Tulsa, OK, USA).

Rhythms that are uniquely identified with environmental factors are marked with different fonts in the daily band. For brevity, let us write them as follows: *C*—circadian rhythm (24.4 h period, bold font), *S*—structural rhythm (31 h period, italics), *T*—tidal rhythm (12.4 h period, underscore). For comparison, their amplitudes are shown in Table 3. 

We consider the *S* rhythm (and rhythms close to it) to be caused by the interaction with structure-forming factors of the hydrological water area (seiches, upwelling, rip currents, etc.) and biological origin (shoals of predators). In fact, this refers to those marine manifestations that are described in works [43,44,45] as submezoscale. It is generally accepted that due to their small spatial scale, the role of such structures in the distribution of zooplankton is quite difficult to identify in situ. Nevertheless, in the conditions of a stationary bioindication station, these synchronizations are quite easily observed. 

The endogenous nature of the *C* rhythm is described in many sources, in particular in [32,39]. This kind of rhythm is determined by the well-known daily vertical migration of zooplankton (diel), which is a universal phenomenon in all oceans of the world as well as in fresh waters. The normal pattern involves the movement from shallow depths at night to deeper depths during the day and vice versa. This allows plankters that feed on phytoplankton and microzooplankton predominantly near the surface, minimizing the risk of predation by fish. It is generally accepted that these movements are synchronized by sunlight; i.e., diel is the manifestation of plankton phototropism [46].

The *T* rhythm is associated with tidal processes [45], which are quite strong in the Barents Sea and obviously lead to plankton advection. Here, the determining factor of the medium for the generation of the *T* rhythm is a tidal wave with a characteristic time of about 12.4 h, which is easily established by the pressure profile (Figure 6) and is associated with the Moon’s influence. Note that in the range of seasonal rhythms, there is a rhythm with a length of 317 h, which corresponds to the period of syzygial tides on the pressure profile in Figure 6, when the tidal forces of the Sun and the Moon are combined. 

Ultradian rhythms in the intra-day range [16,34,43] are quite interesting for the bioindication analysis of ecosystems due to their short period and the possibility of using the Fourier analysis at small time intervals. 

A large number of these rhythms indirectly characterize the biodiversity presented in Table 1. 

The infradian rhythmic processes of the seasonal range in these experiments are also partially registered. To use this correctly, it is necessary to increase the duration of the time series of measurements, for example, to ensure continuous monitoring for a year or more, which is only possible at a fixed monitoring station. 

It should be noted here that the data given in this section with respect to the rhythm set by the time series of long measurements show only the presence of this rhythm and the stability of its manifestation even with a large time averaging. In other words, these data provide qualitative rather than quantitative analysis of the Fourier spectrum of the time series of plankton concentrations in the DHC working volume. Nevertheless, information on the frequency values of particular rhythms can identify the reasons that caused certain desynchronizations on various time scales. 

### 4.3. Circadian Rhythm Amplitude of the Time Series of Plankton Concentration 

In our previous work [16], we measured the rhythmic processes of plankton in Lake Baikal. We also found the daily circadian rhythm during the expedition in high latitudes and in rich biocenosis at the moorage place in the Barents Sea. Indeed, in high latitudes, we are dealing with factors of polar night and polar day that appear in the specific upper illuminance of the photic layer. But this does not lead to a complete absence of the circadian rhythm in the Fourier spectra of the polar plankton concentration series in the DHC working volume, since the “biological clock” does not fail. Another feature of high latitudes is that the amplitude of the circadian rhythm is smaller than the amplitude of the *T* rhythm. In addition, the possibility of diagnosing an ecosystem by the circadian rhythm is determined by the distinguishability of the circadian *C* rhythm against the background of a strong *T* rhythm. 

From the entire rhythm of planktonic biocenosis, we distinguish the circadian rhythm in the bioindication task because of the following:

- There is information on all the connections of the active components of biocenosis, including the synchronizing factors of the medium, in the corresponding structure of the Fourier spectrum of the polar plankton concentration series in the DHC working volume;
- This is an element of self-organization of a large number of individuals of an endogenous nature and is realized with the participation of their nervous system (organs of vision that ensure photosensitivity);
- This rhythm is always present in any water area.

It is these properties that make the circadian rhythm the most acceptable for bioindication. To this end, the ecosystem function ${I_{c}}^{0}\left(j\right)$ can be built, which we defined by the relation (6) as the amplitude of the diurnal circadian rhythm registered on the *j*-th day. Since the function ${I_{c}}^{0}\left(j\right)$ is built by dividing the time sequence describing the concentration of plankton in the DHC working volume into several local regions, it serves the local harmonic interpolation of the time series.

**Figure 7:**
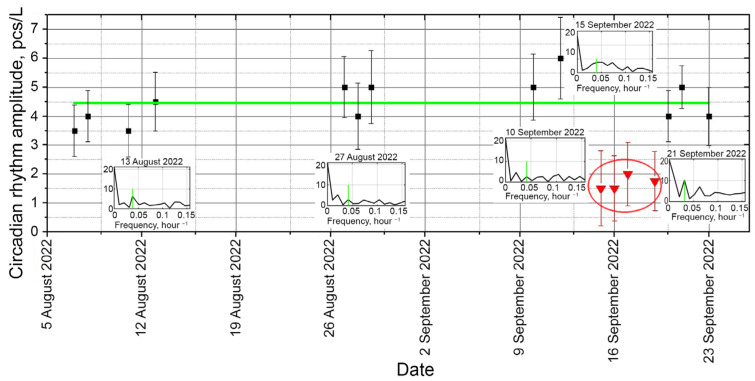
Circadian rhythm of plankton concentration in the DHC working volume for the time series shown in Figure 5a. Green line—interpolation of the circadian rhythm amplitude, the daily readings—black dots and red dots during a rhythm failure under the influence of an indicator impurity. Red circle—time of indicator impurity introduction into biocenosis. Vertical green dashes—manifestations of the circadian rhythm in the daily Fourier spectrum of the time series of plankton concentration in the DHC working volume.

This local approximation allows following a trend without referring to the previous measurements, which is very important for detecting desynchronosis associated with pollution. Thus, the introduction of an indicator impurity was clearly recorded as the disappearance of the circadian rhythm line (Figure 7). 

The introduction of saturated salt solution as a controlled “pollutant” in the plankton habitat from 15 September to 21 September 2022 clearly disrupted both the amplitude and the nature of the spectrum near the circadian rhythm. 

The data on the change in biocenosis due to the change in the medium were received from the host computer a day after the beginning of the experiment (it took so long to form a circadian rhythm line). This is an estimate of the response time of the “zooplankton + DHC” biohybrid sensor system to pollution. 

The rhythm resumed when the impact of the “pollutant” ended. This can be explained by the short time duration of impact in a limited volume (vicinity of the DHC working volume). 

This paper shows the results of measurements taken at a single point in the water area during a long time. Let us find out the conditions under which it is possible to extend the results obtained during the analysis of these data to the entire water area. 

The transfer of impurities in a turbulent flow occurs under the influence of averaged and pulsating components [47]. Turbulized fluid is characterized by self-organization as the process of emergence and complication of mesoscale coherent vortex structures [48,49,50,51,52]. 

In accordance with this self-organization of the flow, the pulsation component generates large relatively ordered vortex structures absorbing the suspension, including plankton, due to a change in the direction of the velocity vector. There are quite a few publications on the interaction of mesozooplankton and these vortices [53,54,55,56,57,58,59,60,61], which show that this interaction is non-linear and affects such plankton processes as nutrient absorption, grazing, predation, and reproduction. However, it is well known that the fine and mesoscale turbulent structure of water masses combined with plankter grazing by predators leads to a characteristic spot-like nature of plankton spatial distributions, which is called the mosaic or fractal structure of biocenoses [62,63]. If the pulsation component of the flow contributes to the diffusion of plankton by carrying it in a vertical plane, then the averaged component ensures the main movement of the flow, as a result of which water masses are transferred along with plankton — advection. 

These plankton movements represent a time sequence of plankton concentration in the recording volume of the measuring device (in this case, the DHC), which is fixed at a certain water area point during a long moorage. Thus, the flow turbulence is one of the compelling reasons for self-oscillations of plankton concentration at such a stationary bioindication station that leads to the S rhythm. The presence of S rhythms in the Fourier spectrum of the diurnal band (Table 2) during monthly sampling suggests that these factors are quasi-regular, as they are caused by the pulsating turbulence of various scales [64,65]. Therefore, on the one hand, the quasi-regularity of coherent vortices leads to quasi-regular pulsations of plankton concentration in the working volume, the period of which is determined by the frequency of the vortices. On the other hand, it appears that by measuring the concentration of plankton as a time series over a certain time $t_{av}$, we achieve the same as performing a number of measurements during spatial scanning (or due to advection) of an extended area of interest (water area) $L_{av}$ by a small measuring volume of the device, as is typically performed in oceanography. 

Thus, if the S rhythm is present in the long time sequence of measurements of plankton concentration in the DHC working volume, then the measurement results can be extended to a certain water area. 

To determine the size of this water area, we have to choose $t_{av}$ so that after averaging the data, the S rhythm disappears in the time series during this time (in fact, this means that this time is sufficient to transfer plankton from one spot of the fractal structure to another). In this case, we are dealing with averaging sufficient to determine a valid average concentration in the area at which spatial scale can be determined from the ratio $L_{av}=t_{av}\cdot u$, where u—velocity of the averaged flow (or flows) in the water area and equal to the velocity of plankton advection. The elimination of S rhythms on the time series in this way is a tool for establishing the correspondence between the time parameters of plankton and the spatial scales of the water area [66]. 

Such estimates can be useful for planning a reference grid, placing a bioindication complex relative to a potential source of pollution, and addressing the problem of pollution propagation through correlation measurements performed using several sensors.

## 5. Conclusions

Thus, the studies performed in natural conditions in the open water area using a submersible digital holographic camera allow for the following conclusions:

- A submersible digital holographic camera (DHC) can be used to detect rhythmic processes associated with plankton;
- In high latitudes, the circadian rhythm of plankton may have anomalies associated with photometric manifestations of “polar night” or “polar day”, which reduce the amplitude of this rhythm in the Fourier spectrum of the time series of plankton concentration in the DHC working volume,
- The ecosystem function, which we called the amplitude of the daily circadian rhythm of plankton, shows bioindicative informativity, which makes it possible to use it to mark the disturbance of the habitat comfort due to the appearance of contamination factors abnormal for this biocenosis, and the relevant response time is 1 day;
- The amplitude of the diurnal circadian rhythm as a bioindication function can be used without considering the specification of certain taxa despite the biodiversity of the water area.

## Figures and Tables

**Table 1:** Zooplankton biodiversity of the Zelenetskaya Bay epipelagial. The scale ruler size in all images shown in the table is 500 μm.

Taxon	Proportion of the Total Concentration According to the Net Data, %	Images of Plankton Individuals Reconstructed from Holograms Recorded by the DHC
Copepoda *Oithona similis*	66.3–90.6% (on average 75.6 ± 2.8%)	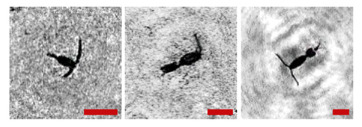
nauplii Copepoda	2.1–4.8% (on average 3.8 ± 2.1%)	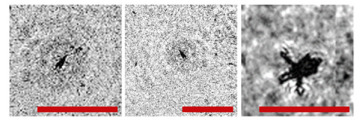
Cladocera *Evadne nordmanni*	2.8–15.4% (on average 8.6 ± 1.6%)	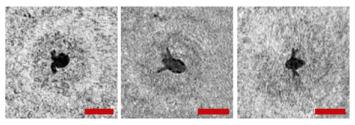
Appendicularia *Fritillaria borealis*	0.4–8.4% (on average 2.4 ± 1.0%)	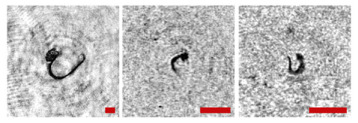
Other	6.2–18.6% (on average 13.3 ± 1.5%)	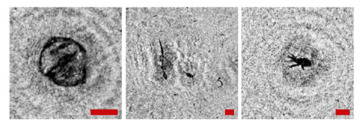

**Table 2:** Statistically significant rhythms of plankton concentration.

Moorage Place	$\mathrm{Seasonal}\;\mathrm{Rhythms}\;f^{-1}$, h	$\mathrm{Diurnal}\;\mathrm{Rhythms}\;f^{-1}$, h	$\mathrm{Intra}\text{-}\mathrm{Day}Rhythmsf^{-1}$, h
Barents Sea, summer	952; 317, 105; 73; 60	45; 39; *31*; 29.8; **24.4**; 22.7; 20.3; 19; 16.7; 16.1; 14.2; 13; 12.4; 11.3; 11.06; 10.7; 10.3; 8.7	8.0; 7.8; 7.2; 6.7; 6.3; 6.0; 5.4; 5.2; 5.1; 5.0; 4.7; 4.1; 4.0; 3.9; 3.5; 3.36; 3.3; 3.2; 3.1; 2.9; 2.67; 2.2

**Table 3:** Amplitudes of characteristic rhythms of plankton of the daily range and the normalization to the amplitude of the circadian (C) rhythm, averaged over the moorage duration.

Moorage Place	${f^{-1}}_{S}$, h^−1^	$c({f^{-1}}_{S})$, ssp/dm^3^	$\frac{c\left({f^{-1}}_{S}\right)}{c\left({f^{-1}}_{C}\right)}$	${f^{-1}}_{C}$, h^−1^	$c({f^{-1}}_{C})$, ssp/dm^3^	${f^{-1}}_{T}$, h^−1^	$c({f^{-1}}_{T})$, ssp/dm^3^	$\frac{c\left({f^{-1}}_{T}\right)}{c\left({f^{-1}}_{C}\right)}$
Barents Sea, summer	31	0.2	0.45	24.4	0.44	12.4	0.7	1.6

## Data Availability

Dataset available on request from the authors.
